# The Boomerang Effect of Suppression of Emotional Expression: Relationship Power, Affectivity and Adolescent and Youth Male-To-Female Dating Violence

**DOI:** 10.1007/s10964-023-01854-y

**Published:** 2023-09-16

**Authors:** Silvia Ubillos Landa, Sandra Nieto González, Alicia Puente Martínez, Marcela Gracia Leiva, José Luis González Castro

**Affiliations:** 1 Faculty of Health Science, University of Burgos, C/Paseo de los Comendadores, Hospital Militar, 1, 09001 Burgos, Spain; 2 Faculty of Social Sciences, University of Salamanca, Campus Miguel de Unamuno, P.° Francisco Tomás y Valiente, s/n, 37007 Salamanca, Spain; 3 Health Education Foundation. Fundadeps, C. de la Costa Brava, 50, 28034 Madrid, Spain; 4 Faculty of Education, University of Burgos, C/ de Villadiego, s/n, 09001 Burgos, Spain

**Keywords:** Dating violence, Perpetration, Power, Affectivity, Suppression of Emotional Expression

## Abstract

Scarce research has been performed on the role of power, affectivity, and suppression of emotional expression in the use of dating violence by adolescents and young men. This study aims to analyze a model of the associations between perceived power (control and dominance), affectivity (positive and negative affect), suppression of emotional expression and the frequency of use of male-to female dating violence. Participants in this cross-sectional and correlational study were 786 Spanish students aged between 13 and 25 years (*M* = 18.80; *SD* = 2.93) divided in two groups: 13–18 (316 adolescents, *M* = 15.58; *SD* = 1.02) and 18–25 (462 young men, *M* = 20.79; *SD* = 1.98) with 8 participants not stating their age. Different sequential mediation models confirmed that, only in young men, affectivity (negative and positive affect) and suppression of emotional expression mediate the relationship between power and the use of dating violence. Fostering equal relationships, associating them with positive emotional states, avoiding the frustration derived from low power perception, and providing young men with strategies for appropriately expressing their emotions may help decrease the use of dating violence.

## Introduction

Violence in dating couples constitutes a serious social and health problem with high prevalence rates among young people (Ybarra et al., 2016). Despite this, most research has focused on studying violence among adults, paying less attention to this type of violence in adolescent and young couples (Jennings et al. 2017). Adolescence and young adulthood (from 10 to 25 years of age) are periods in which people tend to engage in their first dating relationships. Although these relationships are important for their personal and social development, they may also involve strains and conflicts, which may in turn lead to dating violence towards one’s partner (Wincentak et al., 2017). This dynamic implies power imbalances in the relationship and the regulation of affects derived from these tensions and conflicts. Power in relationships is particularly significant during adolescence when status hierarchies are ubiquitous and influence psychological adjustment (Schacter et al., 2023). Likewise, many studies have focused on the role of negative affectivity in predicting dating violence (Armenti & Babcock, 2018). Therefore, this study examines the role of power (understood as control and dominance), negative and positive affectivity, and the suppression of emotional expression in the perpetration of male dating violence against women in adolescent and young men.

Dating violence differs from intimate partner violence among adults in that it often occurs between young people who do not live or have children together and who have no family, financial or legal attachments to each other (Rubio-Garay et al., 2015). There is broad consensus regarding the fact that this type of violence is different from that which occurs in adult couples (Stith et al., 2004; Viejo, 2014). Certain discrepancies exist regarding how long a couple must have been dating before they can be considered to be in a relationship, with opinions ranging from one day (Harned, 2001) to at least one month (Magdol et al., 1998). However, even if dating relationships are short-lived, they are nevertheless an important part of young people’s social lives.

In Western countries, dating relationships and the initiation of sexual activity are considered normative during the transition to young adulthood (Van de Bongardt et al., 2015). Dating relationships can be influenced by the development period in which they occur; based on evolutionary perspectives, two distinct age groups have been identified: adolescents (aged 13–17) and young adults (aged 18–25) (Ibabe et al., 2020). These groups still, mainly, depend on parental support during adolescence and youth while gaining autonomy within peer and romantic relationships (Smetana et al., 2006). Their romantic relationships may shift from primarily recreational, casual, and short-term (Collins et al., 2009) to increased intimacy and longer duration (Arnett, 2014). Young adults often face new responsibilities and stress factors, such as professional development or job search, which can increase conflicts within their relationships (Meier & Allen, 2009).

Studies on the prevalence of dating violence are scarce and their estimates vary widely. In a recent review, percentages oscillating between 19.9 and 95.3% for psychological violence, between 4.8 and 37% for physical violence and between 1.6 and 43.6% for sexual violence were found (Tomaszewska & Schuster, 2021). The results regarding sex differences in aggression rates are also non-conclusive. Although reciprocal participation in violence has been identified as the most common pattern in dating violence (Foshee & Matthew, 2007), except in the case of sexual violence (Krahé et al., 2015), the extant literature indicates that men tend to perpetrate more serious forms of aggression and women report more adverse physical and psychological consequences of dating violence victimization (Reidy et al., 2016).

### Power (Control and Dominance) and Dating Violence

Different studies have linked dating violence to the theory of gender and power (Giordano et al., 2010). This theory posits that the inequality that exists in a relationship is influenced by gender, which limits women’s control in these situations (Buelna et al., 2009). A male’s “Right”, or in other words, the belief that men are intrinsically worthy of privilege or special treatment, is a key motivating factor for young males in the perpetration of dating violence (Reidy et al., 2015). The main tenets that frame this belief include the acceptance of dating violence by male aggressors, the assumption of traditional gender roles and male superiority and the stress generated by the perceived discrepancy between gender roles (Reidy et al., 2015).

Two different theories have developed to explain the relationship between power and male-to-female violence. The first, based on feminist theory (Disch & Hawkesworth, 2016), holds that power imbalances between men and women may increase violence in intimate relationships. Among men, traditional social norms may emphasize male power and enhance intransigent attitudes, the desire to dominate one’s partner, and the sense of being emotionally disconnected; whereas among women, these norms foster submission and passivity (Eaton & Rose, 2011). Advocates of this viewpoint argue that when power is shared in an intimate relationship and decisions are made by consensus, violence levels are lower; in contrast, if power is held by only one member of the couple, the likelihood of violence increases (Kaura & Craig, 2004). A study involving over 4000 young people confirmed that stauncher justification of dominance in a relationship was associated with more male-to-female violence and greater difficulty recognizing violent behavior towards women as abuse (Díaz-Aguado & Martínez, 2015).

The second perspective holds that the power dynamics in intimate relationships is even more complex, because an individual’s ability to fulfill their fundamental needs and aims depends on the cooperation and continued investment of their partner (Kelley & Thibaut, 1978). This interdependence between the two members of the couple restricts each other’s power. Therefore, men, as well as women, are dependent on their partners, a situation that both bestows power on them and restricts said power (Rusbult & Van Lange, 2003; Simpson et al., 2015). However, the loss of power is considered more challenging for men than for women, since it poses a threat to their masculinity (Overall et al., 2016). There is a large body of empirical evidence attesting to the association between low power perception and more aggression towards one’s partner (Overall et al., 2016). Men use more coercive and aggressive strategies to restrict their female partner’s power, as part of their effort to maintain their authority and dominance in the relationship (Hall & Canterberry, 2011). The use of these strategies may be explained by masculine gender role (or discrepancy) stress, which is generated as the result of the perception that one is unable to live up to ideal male roles, prompting the use of aggression as a means of compensating for one’s self-perceived lack of masculinity (Harrington et al., 2021; Reidy et al., 2015). Aggression provides a clear demonstration of power, toughness and independence, and is therefore an effective way for men to reestablish their masculinity (e.g., Dahl et al., 2015). The centrality of power in masculinity, coupled with evidence showing that a threat to masculinity may prompt aggression as a means of restoring it, suggests that men are more likely to respond aggressively in situations in which they perceive low power levels in their relationships.

### Affectivity and Dating Violence Perpetration

Theories about power have tended to overlook the emotional processes involved in the complete range of violent experiences within a couple (Giordano et al., 2016). An individual’s emotional response to the loss of power may be a relevant factor in understanding violent behavior. Studies on dating violence have mainly focused on the study of negative emotions, particularly anger and jealousy, and studies have shown that negative emotions are linked to the perpetration of intimate partner violence (Armenti & Babcock, 2018; Norlander & Eckhardt, 2005). For example, a meta-analysis found that intimate partner violence was moderately associated with anger, hostility and the internalization of negative emotions (Birkley & Eckhardt, 2015). Likewise, when adolescents and young men feel dominated by close relationship partners, they are more prone to having negative self-views and feeling depressed and anxious (Buist et al., 2017) and therefore more likely to use dating violence to assert themselves by dominating their partner (Díaz-Aguado & Martínez, 2015).

Other studies involving an experimentally induced threat to participants’ masculinity have shown that such a situation led to anger and, in turn, to the use of a range of aggressive responses by individuals as a means of restoring their sense of manhood (e.g. Weaver & Vescio, 2015). Adolescent males have been found to employ strategies such as controlling their partner’s appearance and behavior, cheating and threatening to cheat, and sexual and physical violence in response to losing their power within the relationship (Lopez et al., 2012).

### Emotion Regulation: The Role of Suppression of Emotional Expression

Another fundamental aspect to consider is the ability to regulate one’s emotional states. According to the emotion regulation process model, emotional suppression is viewed as a strategy focused on the response to a stressful event, since it involves modifying the behavioral component of an already activated emotional response (e.g., hiding facial expressions of anger while resolving an interpersonal conflict) (Gross, 2015). In a meta-analytical review, the suppression of emotional expression had an impact on the ability to modify one’s emotional response, with a small to medium effect size (Webb et al., 2012). Moreover, specifically, the suppression of emotional expression was found to be a more effective strategy than either the suppression of the emotional experience itself or the suppression of thoughts about the event that generated the emotion in the first place. It is important to note that even if individuals are able to control the expression of their emotions (behavioral measures), this does not necessarily mean they are suppressing the emotional experience itself (experiential measures), nor does it imply the absence of physiological activation (physiological measures).

However, although the suppression of emotional expression may fulfill important social functions, it is not always an adaptive strategy. For example, an inflexible use of this strategy has been linked to aggressive behavior (Norström & Pape, 2010). In some cases, paradoxically, the use of suppression may increase the experience of negative emotions, interfere in decision-making processes, diminish the quality of interpersonal relationships, increase physiological excitation, and hamper the resolution of difficult situations, thereby increasing the likelihood of aggression (Roberton et al., 2012). This could be termed a “boomerang” effect because while trying to reduce the emotional distress (high negative affect and low positive affect) resulting from low perceived relationship power by suppressing the emotional expression what happens is the opposite; there is no reduction of emotional distress, or even an increase in this distress. Moreover, this effect may lead to a higher frequency of dating violence.

## Current Study

Based on the abovementioned, it is necessary to study and understand how affective and emotional regulation processes underlie the relationship between perceived power and perpetration of male-to-female dating violence, in order to promote more effective preventive strategies. The objective is to analyze, among males, a model of the relation between perceived power (control and dominance), positive and negative affect, the suppression of emotional expression and the use of dating violence in two age groups separately: 13–18 (adolescents) and 19–25 (young adults). The study hypotheses are as follows: Differences are expected between the adolescent and young adult groups, with the latter showing a lower perception of control and dominance, more negative and less positive affect, greater suppression of emotional expression, and greater dating violence than adolescents, because of them taking on new responsibilities and experiencing more stress factors that negatively impact the couple’s relationship (Hypothesis 1); Low perceived control (Hypothesis 2) and dominance (Hypothesis 3) in the relationship will be directly associated with a higher frequency of aggression within dating relationships in the overall sample and both groups, but more strongly in the youth group; Low perceived control (Hypothesis 4) and dominance (Hypothesis 5) in the relationship will be associated with an increase in negative affect and a decrease in positive affect, which in turn will be linked to an increase in suppression of emotional expression, giving rise to a greater frequency in the use of dating violence, especially in the case of young adults; Finally, affectivity and the suppression of emotional expression will mediate the relationship between low perceived relationship power and dating violence; with higher negative and lower positive affect, along with greater suppression of emotional expression, strengthening the relationship between low perceived power and greater frequency of dating violence perpetration, mainly in the group of young adults (Hypothesis 6).

## Methods

### Sample

The initial sample (convenience sampling) was comprised of 956 adolescents and young men. All those who did not meet the following inclusion criteria were eliminated (*N* = 170): 14 were over 25 years of age, 10 were not students, 61 originated from other countries different from Spain, 22 had had or have a same-sex partner, 24 had never had a partner, 30 had been in the relationship for less than a month, 8 lived or had lived with their partner and 1 had fathered a child with the couple. The final sample comprised 786 heterosexual Spanish male students aged between 13 and 25 years (*M* = 18.80; *SD* = 2.93). The mean age for the adolescent group (*n* = 316, 13–17 years old) was 15.88 (*sd* = 1.02) and for the young adults (*n* = 462, 18–25 years) was 20.79 (*sd* = 1.98). Eight participants did not inform about their age.

Almost two thirds (*n* = 498; 63.4%) were at high school; over one quarter (*n* = 211; 26.8%) at university; and 3.1% (*n* = 24) were studying at another type of educational institution, such as a private academy or music conservatory. The remaining 6.7% (*n* = 53) did not answer this question. In the adolescent group, almost all participants were high school students (*n* = 314, 99.4%), and only one was enrolled at a university (0.3%) and another at an educational institution (0.3%). In the young adults’ group, 184 (39.8%) were high school students, 210 (45.5%) university students, 23 (5%) were studying at an educational institution, and 45 (9.7%) did not state their enrollment.

As regards their dating situation, 43.1% (*n* = 339) were dating someone at the time of the study (adolescents: *n* = 100, 31.6%; youth: *n* = 239, 51.7%) and 92.4% said they had dated someone in the past (*n* = 726) (adolescents: *n* = 300, 94.9%; youth: *n* = 426, 92.2%).

The number of relationships ranged between 1 and 20, with 81.7% saying they had dated one (*n* = 259, 33%), two (*n* = 233; 29.6%) or three (*n* = 150; 19.1%) people and 14% claiming to have had four (*n* = 51; 6.5%), five (*n* = 41; 5.2%), or more than six (*n* = 38; 4.9%) different relationships. Fourteen (1.7%) participants did not respond to this question. In the adolescent group (range 1–20): 112 (35.4%) adolescents had one relationship, 90 (28.5%) two, 52 (16.5%) three, 18 (5.7%) four, 20 (6.3%) five, and 20 (6.3%) six or more. Four participants (1.3%) did not state their number of relationships. In the young adults’ group (range 1–10): 147 (31.8%) participants had one relationship, 143 (31%) two, 98 (21.2%) three, 33 (7.1%) four, 21 (4.5%) five, 18 (3.9%) six or more. Two participants (0.5%) did not state their number of relationships.

The mean age at which they first started dating someone was 15.44 years (*SD* = 2.84; range: 6–25 years) in the overall sample and 14.53 (*SD* = 3.39) for the adolescent and 16.05 (*SD* = 2.21) for the young adults.

### Procedure

The study design was cross-sectional, descriptive and correlational. The questionnaire was administered to 10 participants online in a pilot study designed to identify any possible errors and estimate the response time. The final version questionnaires were made available in two formats, with 109 participants (12.8%) completing them online using the Qualtrics platform and 740 participants (87.2%) completing them in pen and paper format. The mean response time was 30–40 min.

The research team, composed by four researchers (psychology and social education students), emailed the headteachers at different schools and universities in Spain to explain the study’s research objectives and ask for their participation. Schools that agreed to participate were phoned to plan and carry out the data collection. In all, 6 universities and 10 high schools agreed to participate. The interested schools informed the parents and gathered informed consent from them. The students who were authorized and agreed to participate in the study voluntarily completed the questionnaire during the schools’ established schedules in all classrooms in pen and paper format. The research team carried out the application. The percentage of participants completing the pen and paper questionnaire (81.5–86.1%) or online, using the Qualtrics platform (12.9–18.5%) was similar in schools, universities and other institutions. In addition, following a snowball method, the researchers shared the link and disseminated the study through social networks (Facebook, Twitter, etc.) and an instant messaging service (WhatsApp). Moreover, they emailed the link to potential participants. To avoid duplicates, the IP addresses of all the computers used were recorded. The research project was approved by the Ethics Committee at the University of Burgos (IR 20/2019). The sample size, variables, initial hypotheses, and planned analyses of this study were pre-registered in the OSF-Open Science Framework (osf.io/4zvxf).

### Measures

Information was collected regarding sociodemographic variables (sex, age, place of birth and education level) and relationship status (sexual orientation, present and past dating partners, duration of the relationship(s), number of relationships, age of first dating relationship, existence of marital ties, and whether or not participants lived or had lived with their partner or had children with them). A behavioral measure was used to ask for participants’ sexual orientation; they had to indicate the sex of their most conflictive current or past partner (female, male or other). Next, a battery of different questionnaires was administered. Before responding to scales, participants were instructed to answer all questions in relation to their most conflictive dating relationship, current or past.

#### Dating violence

Cuestionario de Violencia entre Novios (CUVINO-R)/ Dating Violence Questionnaire (DVQ-R) (Rodríguez-Díaz et al., 2017). This instrument comprises 20 items designed to assess five dimensions of violence perpetration among young dating couples (*α* = 0.851; adolescent: *α* = 0.898; young adults: *α* = 0.805). Psychological violence (*α* = 0.768; adolescent: *α* = 0.804; young adults: *α* = 0.741), comprised by: Detachment (attitudes and feelings of indifference towards one’s partner; e.g., “You have ignored their feelings”); Humiliation (making comments designed to undermine their self-esteem and personal pride; e.g., “You ridicule the way they express themselves”); and Coercion (pressuring them through threats or manipulation to overcome their will and control their behavior; e.g., “You talk about the relationships you imagine they have with other people”). Physical violence refers to actions such as hitting them or damaging a personal possession to which they attach value; e.g., “You have hit your partner” (*α* = 0.853; adolescent: *α* = 0.918; young adults: *α* = 0.687). Sexual violence refers to unwanted sexual behaviors; e.g., “You insist on touching them even though you know they don’t like it” (*α* = 0.773; adolescent: *α* = 0.805; young adults: *α* = 0.745). Items are rated on a 5-point Likert-type response scale ranging from 0 (*never*) to 4 (*almost always*). The sum of items was calculated. Higher scores indicate a greater frequency of dating violence.

#### Control and dominance

Sexual Relationship Power Scale (SRPS-M) (Pulerwitz et al., 2000). A reduced version comprising 19 items was used. Items were divided into two subscales (*α* = 0.880; adolescent: *α* = 0.890; young adults: *α* = 0.873): Control in the relationship, comprising 12 items rated on a 4-point Likert-type scale ranging from 1 (*strongly agree*) to 4 (*strongly disagree*) (e.g., “I feel trapped or stuck in our relationship”) (*α* = 0.904; adolescent: *α* = 0.927; young adults: *α* = 0.885); and Dominance in decision-making, comprising 7 items rated on a 3-point Likert-type scale (1 = *Your partner*, 2 = *Both of you equally*, and 3 = *You*) (e.g., “Who usually has more say about whose friends to go out with?”) (*α* = 0.602; adolescent: *α* = 0.677; young adults: *α* = 0.539). Total scores were calculated following the formula proposed by the scale’s original authors (Pulerwitz et al., 2000). Higher scores indicate greater control and dominance. To test the degree of similarity between these two constructs (control and dominance) and coercion (within the psychological component of dating violence), the magnitude of their association was examined. Results showed that coercion is not significantly associated with dominance (*r* = −0.053). Although the relationships between coercion and control (*r* = −0.248, *p* ≤ 0.001) and between dominance and control (*r* = 0.279, *p* ≤ 0.001) are significant, they are deemed as small effect sizes (small: *r* = 0.10; medium: *r* = 0.30; large: *r* = 0.50) (Cohen, 1988). Therefore, they can be considered as not overlapping measures.

#### Positive and negative affect

Positive and Negative Affect Scale (PNA) (Bradburn, 1969). This scale reflects respondents’ mood in relation to their intimate relationship. It comprises 18 items divided into two subscales: positive affect (PA), with 9 items (e.g., “Have you felt that things turned out the way you wanted?”) (*α* = 0.853; adolescent: *α* = 0.858; young adults: *α* = 0.840); and negative affect (NA), with 9 items (e.g., “Have you felt annoyed by your partner or ex-partner?”) (*α* = 0.830; adolescent: *α* = 0.833; young adults: *α* = 0.811). Items are rated on a Likert-type scale ranging from 1 (*never or hardly ever*) to 4 (*almost all the time*). The mean was calculated by adding up all scores and dividing the total by the number of items. Higher scores indicate greater positive and negative affect.

#### Suppression of emotional expression

Measurement of Affect Regulation Styles (MARS) (Larsen & Prizmic, 2004; adapted to the Spanish population by Puente-Martínez et al., 2018). This scale measures respondents’ mood or the emotional intensity of an experience and how they cope with it (in this case, referring to conflict situations with their intimate partner). Two items were used to measure the suppression of emotional expression (“I tried to not let my feelings show, to suppress any expression” and “I faked, or expressed emotions opposite to those I was feeling”) (*α* = 0.779; adolescent: *α* = 0.765; young adults: *α* = 0.787). Response options ranged from 0 = never to 6 = always. A mean score was calculated where higher scores indicate a greater use of these coping and emotion regulation strategies. Following the recommendations of Eisinga et al. (2013) regarding how to determine the reliability of a two-item scale, the Spearman-Brown coefficient also was applied (α = 0.635; adolescent: *α* = 0.631; young adults: *α* = 0.630).

### Analysis Plan

The SPSS statistical package (version 25) was used. Descriptive analyses (frequencies, percentages, means and standard deviations) were carried out to describe the sample and determine the prevalence of dating violence perpetration. Only to determine the prevalence of dating violence, these scores were transformed into a dummy variable (0 = no perpetration versus 1 = perpetration) based on the “zero tolerance” criterion (when the person answered “never” in all the items on the scale, it became 0 and when they wrote 1 or more in any of the items on the scale, it became 1). The rest of the analyses (correlations and mediations) were performed using the dating violence variable as continuous.

To calculate the differences between both groups (adolescents and young adults), Student’s t-tests for independent samples were applied (Hypothesis 1). Preliminary analyses revealed that age was associated with all the variables under study (*p* ≤ 0.050). The procedure used to administer the questionnaire (online or pen and paper) was associated with significant differences in the variable suppression of emotional expression (higher scores in the pen and paper format; *p* ≤ 0.001). Consequently, age (only for the overall sample) and procedure (for the overall, adolescents and young adults’ samples) were included as covariables in the analyses (correlations and mediations). Partial correlations were carried out to analyze the relationship between variables, considering both groups (Hypothesis 2 and 3).

Six multiple sequential mediation analyses were also carried out, one for each dimension of power in the dating relationship (control and dominance) for the overall sample, and then for each age group separately (adolescents and young adults). These analyses were performed using the PROCESS MEDIATE macro for SPSS 25 (Hayes, 2018), with the aim of testing: 1) the direct relationship between the model variables (control and dominance, positive and negative affect, suppression of emotional expression and dating violence) (Hypothesis 4 and 5) and, 2) the indirect effects of control and dominance on dating violence through negative and positive affect (primary mediator: M1) and the suppression of emotional expression (secondary mediator: M2) (Hypothesis 6). The specific indirect effect of each condition (e.g., control) was estimated, while controlling for the other (e.g., dominance) (Model 80). The PROCESS macro estimated the direct, total and indirect effects, along with the standard errors (SE) and confidence intervals (95% CI) based on the distribution obtained using the percentile Bootstrap method. This method uses re-sampling with replacement, generating a series of simulated samples from the original sample in order to calculate the standard error (10000 samples are extracted). The indirect effect is deemed significant if the confidence interval does not exceed zero (Hayes, 2018). The significance level was established at *p* ≤ 0.05.

## Results

### Types and Prevalence Rates for Dating Violence Perpetration

When calculating prevalence rates, the zero-tolerance criterion was applied. In other words, a positive response to any question on the scale was deemed to indicate dating violence perpetration. The most prevalent types of violence in dating relationships were psychological, followed by sexual and physical violence. The global percentage of dating violence was 69.8%. Prevalence rates of all types of dating violence are higher in the young adults group compared to adolescents. Chi-square tests show that there is a significant association between age and dating violence (χ^2^_(778)_ = 22.248, *p* ≤ 0.001), specifically with psychological dating violence (χ^2^_(778)_ = 22.961, *p* ≤ 0.001) and its dimensions (Detachment: χ^2^_(778)_ = 15.316, *p* ≤ 0.001; Humiliation: χ^2^_(778)_ = 9.912, *p* ≤ 0.001; Coercion: χ^2^_(778)_ = 8.469, *p* = 0.004), but not physical and sexual behaviors (Table 1).

**Table 1:** Prevalence rates for dating violence perpetration

	Overall Sample (*n* = 786)				13–17 years (*n* = 316)				18–25 years (*n* = 462)			
Dating violence	YES		NO		YES		NO		YES		NO	
	*N*	*%*	*N*	*%*	*N*	*%*	*N*	*%*	*N*	*%*	*N*	*%*
Psychological	541	68.8	245	31.2	186	58.9	130	41.1	347	75.1	115	24.9
Detachment	474	60.3	312	39.7	163	51.6	153	48.4	303	65.6	159	34.4
Humiliation	129	16.4	657	83.6	36	11.4	280	88.6	92	19.9	370	80.1
Coercion	314	39.9	472	60.1	106	33.5	210	66.5	203	43.9	259	56.1
Sexual	83	10.6	703	89.4	29	9.2	287	90.8	53	11.5	409	88.5
Physical	45	5.7	741	94.3	16	5.1	300	94.9	28	6.1	434	93.9
Total Dating Violence	549	69.8	237	30.2	190	60.1	126	39.9	351	76	111	24

### Differences in Power, Affect, Suppression of Emotional Expression, and Dating Violence between Adolescents and Young Adults

As shown in Table 2, the young adults group (18–25 years old) shows a higher frequency of use of dating violence, less dominance, greater negative and positive affect, and greater suppression of emotional expression than the adolescent group (13–17 years old). Effect sizes are small.

**Table 2:** Mean differences in the use of dating violence, control, dominance, affectivity and the suppression of emotional expression in adolescents and young adults

	Range	Total		13–17 years *n* = 316		18–25 years *n* = 462				
		*M*	*SD*	*M*	*SD*	*M*	*SD*	*t*	*p*	*d*
Dating violence	0–80	3.02	4.62	2.57	5.11	**3.32**	4.27	−2.14	0.017	4.63
Control	1–4	3.17	0.64	3.21	0.70	3.13	0.60	1.66	0.098	0.64
Dominance	1–4	2.41	0.35	**2.46**	0.36	2.37	0.35	3.41	0.001	0.35
Positive Affect	1–4	2.71	0.62	2.62	0.68	**2.78**	0.56	−3.54	0.0001	0.61
Negative Affect	1–4	1.89	0.56	1.74	0.56	**1.99**	0.54	−6.36	0.0001	0.55
^1^SEE	0–6	1.99	1.55	1.81	1.51	**2.10**	1.57	−2.54	0.011	1.54

Levene’s tests were significant in dominance and positive affect

Bolds have been used to indicate which group (among adolescents and young men) had a higher mean

*p* ≤ 0.05 is significant

^1^*SEE* Suppression of Emotional Expression

### Correlations between Dating Violence Perpetration, Dominance, Control, Affectivity and Suppression of Emotional Expression

The use of dating violence was negatively and significantly associated with perceived control and positive affect, but not with dominance. Negative affect and the suppression of emotional expression correlated positively with dating violence. All the above associations had a small effect size. Both control and dominance were negatively and significantly associated with negative affect and the suppression of emotional expression, and positively and significantly with positive affect. All these associations had a small effect size, except for that between control and negative affect and positive affect, which had a medium effect size. Higher negative affect and lower positive affect were associated with greater suppression of emotional expression, with a medium and small effect size, respectively. Finally, control correlated positively and significantly with dominance, and negative affect contrarily and significantly with positive affect with small effect sizes in all cases (see Table 3).

**Table 3:** Correlations between dating violence perpetration, dominance, control, affectivity, and suppression of emotional expression, controlling for procedure in the adolescents and young adults’ groups

	1	2	3	4	5	6
1. Dating Violence	–	−0.195^***^/−0.312^***^	0.122^*^/−0.142^***^	−0.053/−0.182^***^	0.210^***^/0.269^***^	0.134^**^/0.243^***^
2. Control	−0.257^***^	–	0.068 /0.431^***^	0.316^***^ /0.409^***^	−0.167^**^/−0.455^***^	−0.050/−0.257^***^
3. Dominance	−0.027	0.268^***^	–	0.17 /0.299^***^	−0.073/−0.325^***^	−0.076/−0.193^***^
4. Positive Affect	−0.109^**^	0.358^***^	0.161^***^	–	0.135^*^ /−0.319^***^	0.000 /−0.249^***^
5. Negative Affect	0.248^***^	−0.321^***^	−0.221^***^	−0.095^**^	–	0.290^***^/0.408^***^
6. Suppression^a^	0.198^***^	−0.169^***^	−0.151^***^	−0.129^***^	0.366^***^	–

The data below the diagonal corresponds to the total sample, and the data above the diagonal to the data by age group (adolescent/youth); Age and procedure are controlled for the total sample and only the procedure in the adolescent and youth groups

^a^Suppression of Emotional Expression

*p ≤ 0.05; **p ≤ 0.010; ***p ≤ 0.001

According to the age group, young adults’ results are identical to those obtained with the overall sample, except the relationship between dating violence and dominance is now negative and significant. Associations between control and dating violence, dominance, positive and negative affect, dominance and negative affect, positive and negative affect, and negative affect and suppression of emotional expression have a medium effect size. However, the data in the adolescent group differ notably. The relationships between frequency of dating violence and positive affect, control and dominance, control and suppression of emotional expression, dominance and negative and positive affect, dominance and suppression of emotional expression, and positive affect and suppression of emotional expression are no longer significant. As in the young adults’ group, the relationship between frequency of dating violence and dominance is also positive and significant. Moreover, negative affect is positively associated with positive affect, in contrast to young adults. In adolescents, all the above associations have a small effect size, except for that between control and positive effect, which has a medium effect size.

### Affectivity and Suppression of Emotional Expression as Mediators between Control and Dominance and Dating Violence Perpetration

**Fig. 1:**
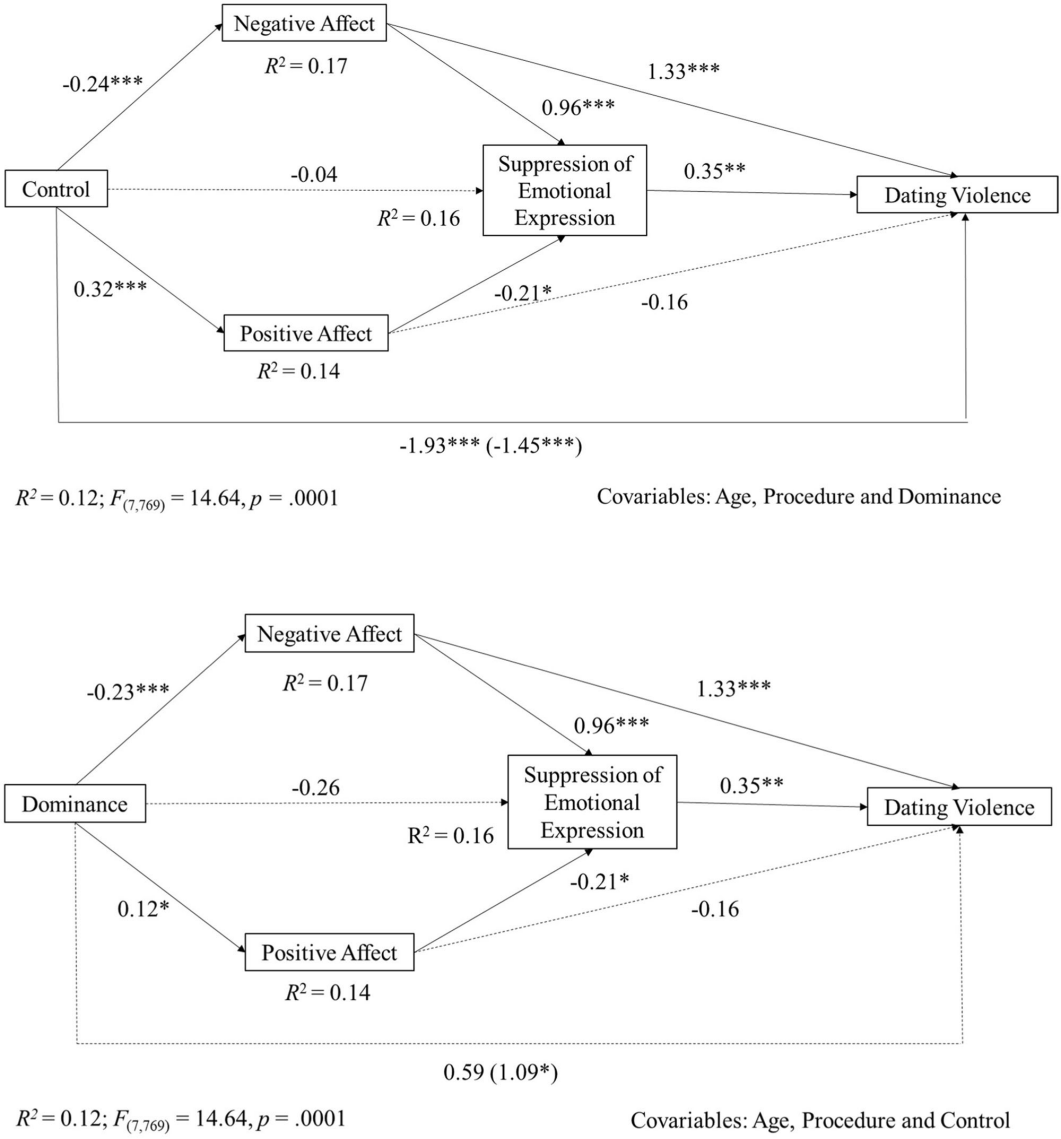
Multiple mediation model. Indirect effect of control and dominance on dating violence through affectivity (negative and positive affect) and the suppression of emotional expression with the total sample. Note. The figures show non-standardized regression coefficients (B). Coefficients pertaining to direct effects are shown in parentheses. Continuous lines represent the effects of the hypothetical model; dotted lines represent non-significant coefficients in the hypothetical model. *p ≤ 0.05. ** *p* ≤ 0.01. *** *p* ≤ 0.001

Considering the total sample, the direct and total effects of control on dating violence were negative and significant. In the dominance model, direct effect was positive and significant while total effect did not reach significance (see Fig. 1).

In relation to Hypotheses 4 and 5, low perceived control and dominance were associated with an increase in negative affect and a decrease in positive affect, which was associated with more frequent suppression of emotional expression. Moreover, in both models, the associations between control and dominance and the suppression of emotional expression were non-significant; suppression of emotional expression was associated with higher levels of dating violence perpetration and the association between negative affect and dating violence was positive and significant whereas the association between positive affect and dating violence was non- significant.

**Fig. 2:**
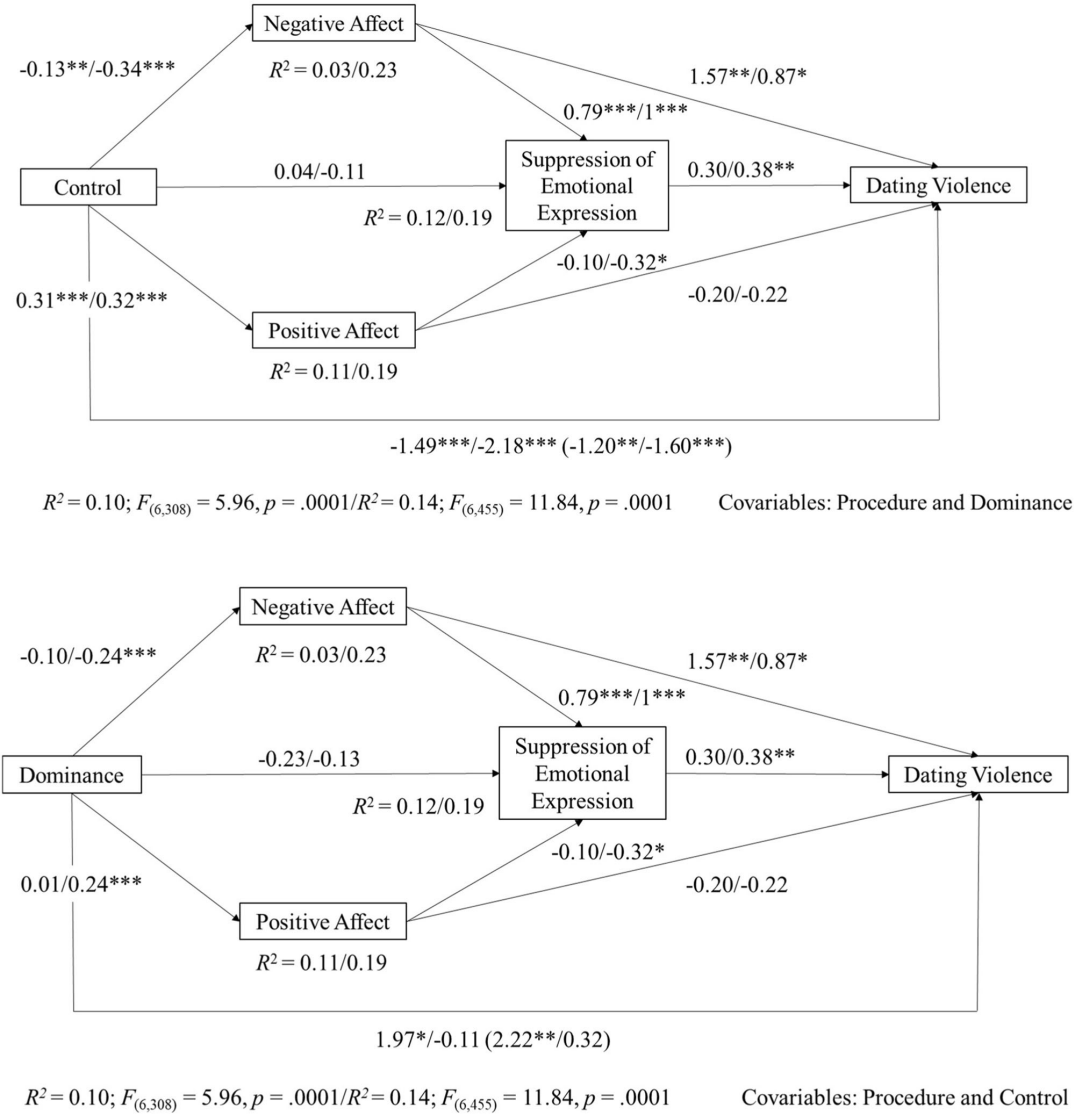
Multiple mediation model. Direct and indirect effects of control and dominance on dating violence through affectivity (negative and positive affect) and the suppression of emotional expression in adolescents and young adults. Note. The figures show non-standardized regression coefficients (B). Coefficients pertaining to direct effects are shown in parentheses. Data show coefficients for adolescent (*N* = 315)/young adults (*N* = 462) groups separately. *p ≤ 0.05. ** *p* ≤ 0.01. *** *p* ≤ 0.001

Results were similar for the age group models (see Fig. 2). However, the relationship between positive affect and suppression of emotional expression and between suppression of emotional expression and dating violence is non-significant for the adolescent group. In the dominance model, the direct and total effect between dominance and dating violence becomes significant in the adolescent group while in the young adults’ model was non-significant. Moreover, findings showed a non-significant association between dominance and positive and negative affect, between positive affect and suppression of emotional expression, and between suppression of emotional expression and dating violence in the adolescent group, while these relationships were significant in the model of young adults’ participants. The rest of the variables in the dominance model showed significant results similar to the overall model.

Upon testing Hypothesis 6, statistically significant indirect effects of control and dominance on dating violence only through negative affect and the serial mediation of negative affect and the suppression of emotional expression were found. Moreover, only in the control model, were there significant indirect effects through positive affect and the suppression of emotional expression on dating violence (see Table 4).

**Table 4:** Specific indirect effects of control and dominance on dating violence in total sample, adolescents and young adults

	Overall sample *n* = 777				Adolescents (13–17 years) *n* = 315				Youth (18–25 years) *n* = 462			
Specific indirect effects (mediators)	*b*	Boot *SE*	Boot 95% CI		*b*	Boot *SE*	Boot 95% CI		*b*	Boot *SE*	Boot 95% CI	
			*LL*	*UL*			*LL*	*UL*			*LL*	*UL*
Control → NA→ DV	**−0.32**	**0.09**	**−0.521**	**−0.150**	**−0.20**	**0.11**	**−0.452**	**−0.042**	**−**0.30	0.20	**−**0.700	0.071
Control → PA→ DV	**−**0.05	0.09	**−**0.228	0.109	**−**0.06	0.12	**−**0.331	0.145	**−**0.07	0.16	**−**0.398	0.237
Control→ ES → DV	**−**0.01	0.03	**−**0.086	0.054	0.01	0.04	**−**0.080	0.103	**−**0.04	0.06	**−**0.172	0.062
Control→ NA→ SEE→ DV	**−0.08**	**0.03**	**−0.147**	**−0.030**	**−**0.03	0.02	**−**0.088	0.000	**−0.13**	**0.06**	**−0.284**	**−0.028**
Control → PA→SEE → DV	**−0.02**	**0.01**	**−0.053**	**−0.002**	**−**0.01	0.01	**−**0.042	0.017	**−0.04**	**0.02**	**−0.092**	**−0.002**
Dominance → NA → DV	**−0.30**	**0.11**	**−0.528**	**−0.118**	**−**0.16	0.16	**−**0.516	0.139	**−**0.21	0.15	**−**0.535	0.049
Dominance → PA → DV	**−**0.02	0.04	**−**0.104	0.042	0.00	0.03	**−**0.071	0.078	**−**0.05	0.13	**−**0.327	0.185
Dominance →ES → DV	**−**0.09	0.07	**−**0.236	0.019	**−**0.07	0.09	**−**0.290	0.049	**−**0.05	0.09	**−**0.248	0.133
Dominance →NA→ SEE → DV	**−0.08**	**0.03**	**−0.149**	**−0.024**	**−**0.02	0.03	**−**0.093	0.023	**−0.09**	**0.05**	**−0.202**	**−0.014**
Dominance →PA → SEE → DV	**−**0.01	0.01	**−**0.026	0.000	0.00	0.00	**−**0.008	0.010	**−0.03**	**0.02**	**−0.077**	**−0.001**

Bolds have been used to indicate which specific indirect effects are significant for each group and for the overall sample

*NA* Negative Affect, *PA* Positive Affect, *SEE* Suppression of Emotional Expression, *DV* Dating Violence

As shown in Table 4, the indirect effects of the control and dominance models in adolescent and young adults vary compared to those of the overall model. Findings show a significant indirect effect from control to dating violence through negative affect in the adolescent group, but not for young adults. In the control and dominance models, results also find significant indirect effects from control and dominance to dating violence through affects (negative and positive) and suppression of emotional expression.

## Discussion

Dating violence among adolescent and young adults is a pervasive problem with harmful effects that last one’s whole life. However, the pattern of relationships between factors such as power and emotional regulation that might explain the use of violent behavior in dating relationships is still unclear. This study addressed prominent gaps in the literature providing empirical evidence of a relationship model between power (desire for dominance and control), affectivity, suppression of emotional expression and the use of violent behaviors in dating relationships by adolescent and young heterosexual males. The findings emphasize the importance of paying attention to those variables that could exacerbate the use of dating violence.

Using the “zero tolerance” approach results found that dating violence was perpetrated by approximately 70% of male participants. Moreover, results confirm a higher use of dating violence, especially of a psychological nature, among young adults than adolescents. Likewise, in a meta-analysis, the rate of sexual violence perpetrated by boys aged between 13 and 18 years was similar to that found in the present study (10 vs. 10.6%), although physical violence was lower (13 vs. 5.7%) (Wincentak et al., 2017). Compared with data from other high-income European countries (Tomaszewska and Schuster, 2021), the prevalence of sexual (10.6%) and physical (5.7%) violence in this study was in the lower range (sexual: 2.6–37%; physical: 4.8–34.9%). However, the psychological rate (68.6%) is higher than that found in these other countries (19.9–42.7%). Nevertheless, in all countries psychological violence is the most common type of violence. This is important since longitudinal studies have confirmed that psychological violence is a good predictor of physical aggression in both the present and future (O’Leary & Smith Selp, 2003), and that physical violence exacerbates psychological aggression (Antônio & Hokoda, 2009), thereby confirming the existing relationship between different types of violent behavior.

In addition to socioeconomic and cultural aspects, it is important to note that prevalence rates of dating violence are also sensitive to a range of different factors, including a) the definitions used; b) participant selection; c) data sources and collection instruments; d) operationalization of the variables; e) the time period covered by the assessment; f) statistical processing of the data; and g) social desirability bias and participants’ willingness to disclose sensitive information about their lives (Krug et al., 2002). In this study, the use of a “zero tolerance” approach, firstly, allowed comparisons of the rates of violence with previous studies (Ibabe et al., 2016; Reidy et al., 2015). Secondly, dating violence is a serious problem with significant consequences for physical and mental health, social and emotional development (Niolon et al., 2019). Thus, it is essential to consider all levels of dating violence within relationships. Studies show that violence within romantic relationships tends to escalate in frequency and severity with age, often starting with low-severity psychological violence (Orpinas et al., 2013; Saint-Eloi Cadely et al., 2020). Likewise, when violence increases, so does the degree of acceptance of their partner’s abuse since continued submission to aggression leads to its normalization (Grest et al., 2020). Third, content analysis of the scale items supports the application of “zero tolerance” since all included behaviors constitute explicit acts of aggression. Moreover, adopting a “zero tolerance” approach sends society a clear message that dating violence is unacceptable (Maquibar et al., 2017), especially as some young people may justify or misinterpret violence due to sexist ideals or a high tolerance for such behavior. This tolerance also hampers the recognition of violence within intimate relationships (García-Díaz et al., 2020). However, results should be taken with some caution since this classification method does not distinguish between different levels or types of violent experience. It includes within the same group (perpetrators) people who have exercised a diverse range of dating violence, ranging from male-chauvinism or micro-sexism and sporadic aggression (a single item) to continuous aggressive dynamics.

Few studies have focused on the role of power (control and dominance) in predicting the use of dating violence by young heterosexual men. The present study makes a significant contribution to this field and touches upon four relevant issues. First, as indicated in hypothesis 1, young males report higher rates than adolescents in the use of dating violence. This result is in line with previous research that found an increase in the use of violence against women by age (Gracia et al., 2023). Moreover, it is consistent with the lower perception of control (although differences are non-significant) and dominance by young men as the use of dating violence would be an attempt to gain power over the female partner (Toplu-Demirtaş & Fincham, 2022). A greater presence of positive and negative affect and a greater use of suppression of emotional expression has also been found in young men, compared with adolescents. Perhaps this is because young males have developed strategies that allow them to maximize the impact of positive emotions (Windsor & Anstey, 2010), although they are also subject to more stressors, which causes them, at the same time, to experience more negative emotions (Coiro, Bettis & Compas, 2017) using suppression of emotional expression as a form of regulation more than adolescents.

Second, a low perception of control is directly associated with a greater use of dating violence in both samples of adolescents and young adults (Hypotheses 2). The relationship between the dominance dimension of the power construct and dating violence is identical in the sample of young adults; however this association is positive in the case of adolescents. In both adolescent and young adults, the result regarding the direct effect of control on the use of violent behaviors is consistent with that reported in a previous meta-analysis (Spencer et al., 2021), which found that, among men, displaying controlling behaviors towards one’s partner was a significant risk marker for the perpetration of physical violence. This finding is also consistent with the gender role stress theory, which views masculinity as a precarious status that requires constant manifestations of its validity. The theory postulates that when men are unable to fulfill the imperatives of the male gender role (exerting power in their romantic relationships) and feel that their masculine identity is threatened, they are more likely to behave violently towards their partner and perceive this aggression as positive and legitimate, since it constitutes a means by which they can restore their status (Vandello & Bosson, 2013). Adolescents and young men may try to control their partners as a means of protecting their maleness (Bosson & Vandello, 2011) and alleviate their stress and feelings of insecurity and personal weakness (Gallagher & Parrott, 2011). Certain problematic male behaviors (such as aggression) may be reinforced because of biased thinking and exaggerated fears within the relationship (Bosson et al., 2006).

However, in the case of young men, the magnitude of the relationship between the control component of power and the use of violence in dating relationships is greater than that of dominance. This result may be because lack of control within a relationship is a proximal variable (to the use of violence) that is associated more directly with aggression (Stark, 2010). Consequently, violence perpetrated within an intimate relationship is not so much a response to a desire to dominate one’s partner as it is a response to a perceived threat to one’s capacity for control. In this case, violence would constitute a tool used by the aggressor to gain more control in the relationship, or to discourage or trigger specific behaviors in their female partner (Toplu-Demirtaş & Fincham, 2022). In a meta-analysis, dominance was found to be linked to violence perpetrated by male abusers, although this association was only moderate and weaker than that found between violence and control (Ubillos Landa et al., 2020). Studies often refer to dominance as power transferred to men by cultural values and beliefs that foster male violence towards women (Moyano & Sierra, 2016). Since dominance implies a fixed and timeless structure (a structural dimension), its influence on intimate partner violence may be more indirect and diffuse (Johnson & Ferraro, 2000).

In the adolescent group, the association between control and dating violence is weaker. Furthermore, contrary to expectations, dominance is positively associated with the frequency of dating violence. This is consistent with a study that found that one of the main predictors of male dating violence against girls was the justification of dominance and the use of aggression to resolve conflicts (Díaz-Aguado & Martínez, 2015). The lack of experience in managing intimate relationships in adolescence leads to important changes in young adulthood (Giordano et al., 2006). In this sense, adolescents may initially be guided more by cultural beliefs about male dominance learned in different socialization contexts. Thus, a greater perception of dominance would be directly associated with a greater use of dating violence. However, as experience increases, young men do not report more power within their relationships, but rather greater attempts to control partners in order to have greater influence over them. Thus, as they gain more relationship experience, the idea of control has an active and grounded quality as it reflects each partner’s attempts to influence or change the other. This more localized focus on control dynamics inevitably connects to specific issues of concern and, at times, dispute that have the potential to further illuminate gender dynamics within early dating relationships (Giordano et al., 2021).

Third, in accordance with Hypothesis 4 and 5, this study found, in the adolescent and young adults’ samples, that the perception of weaker control was associated with high negative affect and low positive affect. In the dominance model, these relationships are significant only in the young adults’ sample. Further, consistent with previous literature, only negative affect was found to positively correlate with dating violence. A previous meta-analysis found an association between negative emotions and dating violence perpetration among young people (Birkley & Eckhardt, 2015) and another study confirmed that negative affect (anger) acted as a mediator between shame and dating violence (Harper et al., 2005). It therefore seems that dating violence is associated more with high negative affect (emotions such as anger, sadness, fear, jealousy) than with low levels of positive affect. Finally, both low positive affect and high negative affect were found to be associated with greater suppression of emotional expression, which in turn was linked to higher levels of dating violence. In the adolescent sample, the relationship between positive affect and suppression of emotional expression was non-significant. Even when individuals were good at suppressing the expression of their feelings, they had trouble eliminating the physiological and experience-based components of their emotions (Quartana & Burns, 2007). Experimental studies have shown that, in accordance with the limited strength model (Muraven et al., 1998), suppressing emotions, particularly negative ones, requires a large amount of effort and energy, leading to the depletion of self-control resources (Hagger et al., 2010). The suppression of emotional expression may exacerbate aggression by exaggerating negative affect, lowering inhibitions against aggression, compromising decision-making processes, diminishing social networks, increasing physical excitation and hampering the resolution of difficult situations (Roberton et al., 2012).

Fourth, the results of the sequential mediation analysis, in line with hypothesis 6, confirmed, only in the case of young adults, that both negative affect and the suppression of emotional expression mediated the relationship between power (both control and dominance) and dating violence. Moreover, positive affect and the suppression of emotional expression also mediated the relationship between control and dominance and violence. In other words, high negative affect and low positive affect and the dysfunctional regulation of these feelings may precipitate dating violence (Cuccì et al., 2019). These results are consistent with the modal model of emotions (Gross, 2015), which stresses that emotions involve an assessment process in which perceptions about the world (low perceived power) are compared with representations about the desired state of the world (male power and hegemony), with the observed discrepancies giving rise to action responses designed to close the perceived gap. In fact, studies on emotion regulation and violence suggest that an inability to effectively manage negative emotions resulting from this perceived gap, such as anger, predicts male violence in young dating couples (Norlander & Eckhardt, 2005; Stith et al., 2004). These results are also consistent with the pyramid model of male aggression which argues that any conflict requires a patriarchal base (unequal social organization that legitimates the desire for dominance), socialization processes, expectations of control within the couple and a trigger event (Bosch-Fiol & Ferrer-Pérez, 2019). It is these individual expectations of control, coupled with a biased perception of events (e.g., interpreting something as calling their masculinity into question, or feeling that the relationship is threatened) that ultimately prompt young men to behave violently towards their partner during a conflict. Moreover, in this pyramid model, emotional intelligence and emotion regulation are key elements for explaining aggression. In other words, although aggressors and non-aggressors are both socialized in accordance with traditional masculinity and have all the other stages of the pyramid in common, non-aggressors reject violence and use more adaptive emotion regulation strategies, whereas aggressors make excessive use of emotional suppression. In the adolescent sample, the proposed model does not work as expected. Only negative affects explain the relationship between low perceived control and the use of dating violence.

Results show that, only in the case of young adults, the attempts to regulate the emotional distress produced by the low perception of power produces the opposite result (boomerang effect). In other words, using the suppression of emotional expression does not manage to reduce the levels of violence towards a partner, but rather they increase. Furthermore, although it is not significant, in adolescents the pattern of results is similar. Perhaps the greater experience that young adults may have in relationships is consolidating this relationship model.

One of the strengths of this study is that it offers a diagnosis of the problem of dating violence among adolescents and young men and further advances understanding of the associated factors that underlie perpetration of violent behaviors. This contribution may help guide intervention efforts designed to prevent and treat the perpetration of dating violence by differentiating between adolescents and young adults. It emphasizes the need to strengthen primary prevention, especially among adolescents, with the aim of reducing the incidence of violence in romantic relationships later in life (Taylor et al., 2013). Prevention should be carried out at an early age (during adolescence) as resistance to any type of intervention tends to be high among young and adult males due to their total or partial refusal to acknowledge the problem (Boira et al., 2014). Moreover, if the aggressive interaction patterns assimilated by adolescents are maintained as they progress to adulthood, they are likely to manifest in the form of much more serious violent behaviors (González-Ortega et al., 2008). This deconstruction can be done through interventions focused on reflecting on the new masculinities, thus providing adolescents with alternative models to those offered by patriarchy (Sims & Rodríguez-Corcho, 2022). On the other hand, and, although they can also be included within primary prevention, it is important in secondary and tertiary prevention to pay attention to the role played by “the need for control”, especially in young adults; the focus should be on working on cognitive restructuring in the presence of distorted cognitions about the lack of control within the relationship and delegitimizing the use of violence as a means to restore the control they believe they have lost and working on teaching alternative behaviors (Rizzo et al., 2018). Likewise, all prevention levels should include content on the use of maladaptive emotion regulation strategies, such as suppression of emotional expression. The use of this coping strategy depletes the ability to implement other more functional coping strategies. Therefore, particularly in young men, it is important to provide resources to enable them to make use of more adaptive coping strategies so that they do not resort to violence as a means of coping.

Despite the findings and practical implications outlined above, the present study has certain limitations, such as, for example, the fact that its cross-sectional design precludes drawing any conclusions regarding causality. Moreover, although the sample group was large, since the sampling technique was non-random, the results should be interpreted with caution and cannot be generalized to other populations. Past studies have observed cultural variations in findings linked to dating violence (Connolly et al., 2010) that were not taken into consideration here as the sample consisted only of Spanish adolescents and young men. It would therefore be interesting for future studies to analyze possible cultural differences in the model of associations proposed here. Another limitation is linked to the fact that this sample comprised only heterosexual dating couples. In the future, researchers may wish to include homosexual dating couples also in order to analyze possible differences in the use of violent behaviors in dating relationships in accordance with sexual orientation. They may also wish to include online violence when dating (i.e., violence perpetrated over the Internet and on social media), since the digital world is one of the main socialization environments for young people. In addition, the extant literature suggests that other individual variables, such as impulsive personality traits, hostility towards women, negative attributions and other emotion regulation strategies not contemplated in the present study may also contribute to explaining the relationship between control, dominance and dating violence perpetration.

## Conclusion

Given the lack of knowledge on the emotional processes that underlie the relationship between perceived power and perpetration of male-to-female dating violence, the present study presents a series of empirical findings that support the role played by relationship power in male-to-female dating violence perpetrated by young heterosexual men and adolescent boys. The results confirm that affectivity, particularly negative emotional states, and the suppression of emotional expression mediate the association between low power perception (control and dominance) and dating violence. Fostering equal relationships among adolescents and young adults, associating them with positive emotional states, avoiding the frustration derived from low power perception, and providing young males with strategies for appropriately expressing their emotions may help decrease the perpetration of dating violence. Given that violence perpetrated by young men is a problem that originates during adolescence, this developmental stage emerges as a critical period for working on the variables outlined above, with a view to preventing the exacerbation and persistence of dating violence during subsequent life phases.

## Data Availability

The datasets from the current study are available at the Open Science Framework repository, https://mfr.osf.io/render?url=https://osf.io/download/amdcp/?direct%26mode=render. The hypotheses, study design, sampling plan, variables, and analysis plan were preregistered in the Open Science Framework repository osf.io/4zvxf.
